# Safety, tolerability, and immunogenicity of INO-4500, a synthetic DNA-based vaccine against Lassa virus, in a phase 1b clinical trial in healthy Ghanaian adults

**DOI:** 10.3389/fimmu.2025.1658549

**Published:** 2025-10-24

**Authors:** Kwadwo Ansah Koram, Kathleen A. Walker, Bonaventure Orizu, Idania Marrero, Jean Boyer, ShuPing Yang, Kate E. Broderick, Kwadwo Asamoah Kusi, Eric Kyei-Baafour, Ebenezer Addo Ofori, Abigail Pobee, Susan Adu-Amankwah, Mary Amoakoh-Coleman, Hannah Brown Amoakoh, Benjamin Abuaku, Edem Badji, Michael Ntiri, Lydia Quaye, Matthew P. Morrow, Albert J. Sylvester, Emma L. Reuschel, Elisabeth Gillespie, David Liebowitz, Laurent M. Humeau

**Affiliations:** ^1^ Department of Epidemiology, Noguchi Memorial Institute for Medical Research, College of Health Sciences, University of Ghana, Legon-Accra, Ghana; ^2^ Inovio Pharmaceuticals, Inc., Plymouth Meeting, PA, United States; ^3^ Department of Immunology, Noguchi Memorial Institute for Medical Research, College of Health Sciences, University of Ghana, Legon-Accra, Ghana; ^4^ Department of Global Public Health and Bioethics, Julius Center for Health Sciences and Primary Care, University Medical Center, Utrecht University, Utrecht, Netherlands

**Keywords:** DNA medicine, Lassa fever, Lassa virus (LASV), safety, immunogenicity, electroporation (EP), vaccine

## Abstract

**Background:**

Lassa fever (LF) is an acute viral hemorrhagic illness endemic to West Africa, with no licensed vaccines or targeted treatments available, highlighting a critical gap in global health preparedness. T cell-mediated immunity plays a central role in viral control and survival. Synthetic DNA vaccines offer a promising strategy to induce both humoral and cellular immunity against LF.

**Methods:**

A Phase 1b, randomized, double-blind, placebo-controlled trial was conducted to assess the safety, tolerability, and immunogenicity of INO-4500, a DNA vaccine encoding the Lassa virus (Josiah strain) glycoprotein precursor (GPC). A total of 220 healthy adults were randomized to receive either 1 mg or 2 mg of INO-4500 (intervention), or placebo, administered intradermally (ID) followed by electroporation (EP) at Day 0 and Week 4. Safety was evaluated through Week 48. Primary immunogenicity endpoints included humoral and cellular immune responses at multiple timepoints post-vaccination.

**Results:**

INO-4500 was well tolerated, with no Grade 3 or higher treatment-emergent adverse events (TEAEs) deemed to be related to the intervention; 88.6% of all TEAEs were Grade 1. No cases of attributable hearing loss were reported. INO-4500 groups demonstrated statistically significant increases in Lassa virus GPC-specific binding antibodies at Weeks 6 and 12 compared to placebo, with the 2 mg group eliciting the strongest responses. T cell responses remained elevated above baseline through Week 48 in both INO-4500 groups, indicating durable cellular immunity.

**Conclusions:**

DNA vaccine INO-4500 was well tolerated and elicited durable humoral and cellular immune responses in healthy adults. These findings support further clinical development of INO-4500 as a potential preventive vaccine to reduce LF-associated morbidity and mortality in endemic regions.

**Clinical Trial Registration:**

https://clinicaltrials.gov, identifier NCT04093076

## Introduction

Lassa fever (LF), a devastating acute viral hemorrhagic zoonotic illness caused by the Lassa virus (LASV), poses a significant public health threat in West Africa (1, 2). An estimated 100,000 to 200,000 infections occur annually due to LASV infection in the region, resulting in approximately 5,000 deaths per year (3, 4). Symptoms range in severity, from mild disease to severe hemorrhagic fever leading to death (5). The disease’s severity is reflected in its case-fatality rate of 1-2% (6, 7), which rises to an estimated 15% among those hospitalized with severe symptoms (4, 7). Pregnant women are particularly impacted by LF, with case-fatality rates up to 30%, along with high rates of neonatal and fetal losses (8). One in five infections result in severe disease, impacting the liver, spleen and kidneys (7), and can include debilitating sequelae such as sensorineural hearing loss, polyserositis, vision distortion, vertigo, and back pain (5).

The absence of approved vaccines or therapeutics targeting LF underscores a critical gap in global health security (4, 5). This void, coupled with LF’s high incidence and severity, poses a significant threat to people living in endemic populations and travelers who visit LASV-endemic areas. The World Health Organization’s (WHO) designation of LASV as a priority pathogen requiring urgent research and development action highlights the need for effective interventions (9). Significant strides have been made in LF vaccine development, with multiple vaccine candidates progressing through clinical trials (4, 10–13). The Coalition for Epidemic Preparedness Innovations (CEPI) is further advancing these efforts by supporting the development of vaccines for priority pathogens such as LASV (9, 14).

Most studies to date, both in human and animal disease models like ours (15), provide evidence that T cell-mediated immunity is key to virus control and survival (15–19), although there is some evidence of non-neutralizing antibodies playing a protective role (5, 6, 20, 21). Potent memory CD4+ T cell responses targeting viral nucleoproteins and surface glycoproteins have been detected in healthy seropositive individuals living in LF endemic areas (19, 22), suggesting the activation of CD4+ T cells in mild and/or asymptomatic infections. Furthermore, in LASV infection, both CD4+ and CD8+ T cell-mediated immune responses are detectable in individuals who ultimately clear the virus, underscoring the essential role of these responses in effective viral clearance and recovery (18, 22, 23). While neutralizing antibodies alone are likely insufficient for protection (24, 25), non-neutralizing binding antibodies such as IgG may contribute to the overall immune defense, as evidenced by their production post-infection (5, 21). More research is needed to confirm these findings and further identify other factors that may contribute to protection against LF.

While the correlates of protection for LF are still being studied, it is currently believed that early virus-specific cellular responses constitute the predominant mode of protection, and are associated with survival, whereas delayed cellular responses are associated with fatal outcomes (5, 16, 18, 26, 27). This insight influences vaccine design strategy, as vaccines that predominantly induce cellular responses are more likely to provide protection against LASV (28).

Preclinical studies for one such vaccine candidate, INO-4500, has yielded encouraging results, demonstrating the presence of LASV-specific memory T cells in vaccinated non-human primates (NHPs) up to one year post-vaccination and conferring both short- and long-term protection against lethal LASV challenge (15). INO-4500 is a synthetic DNA vaccine encoding for the Lassa virus (Josiah strain) glycoprotein precursor (LASV GPC) and is injected intradermally (ID) and followed by electroporation (EP) to enhance cellular uptake. The vaccine targets the GPC protein of LASV, which plays a critical role in host entry, and represents the most conserved region in this genetically diverse virus (29, 30).

We present here the outcomes of a Phase 1b trial (LSV-002) conducted in Ghana, where we evaluated the safety, tolerability and immunogenicity of INO-4500 in healthy volunteers with no known prior exposure to LASV.

## Materials and methods

### Study design and population

LSV-002 was a Phase 1b, randomized, double-blinded within study group, placebo-controlled clinical trial to evaluate the safety, tolerability, and immunological profile of INO-4500 administered by ID injection followed by EP using the CELLECTRA^®^ 2000 ID device (NCT04093076). INO-4500, the active investigational product used in this clinical trial, contains a DNA plasmid designed to express Lassa virus (Josiah strain) glycoprotein precursor. The study was performed in accordance with the principles of the Declaration of Helsinki, Good Clinical Practice (GCP), and applicable regulatory requirements. The study was approved and conducted at the Clinical Trials Unit of the Noguchi Memorial Institute for Medical Research (NMIMR), University of Ghana, Legon.

Healthy adult volunteers, 18–50 years of age, were enrolled at a single location from within and around the University of Ghana community in Accra, Ghana. Written informed consent was obtained from all participants before any protocol procedures were performed. Inclusion criteria included the capability of following study procedures and practicing adequate contraception during the trial period. Exclusion criteria included: participants with a hearing level threshold greater than 30 dB for any frequency tested between 500 Hz – 8000 Hz; pregnant or lactating female participants.

### Study procedures

**Figure 1:**
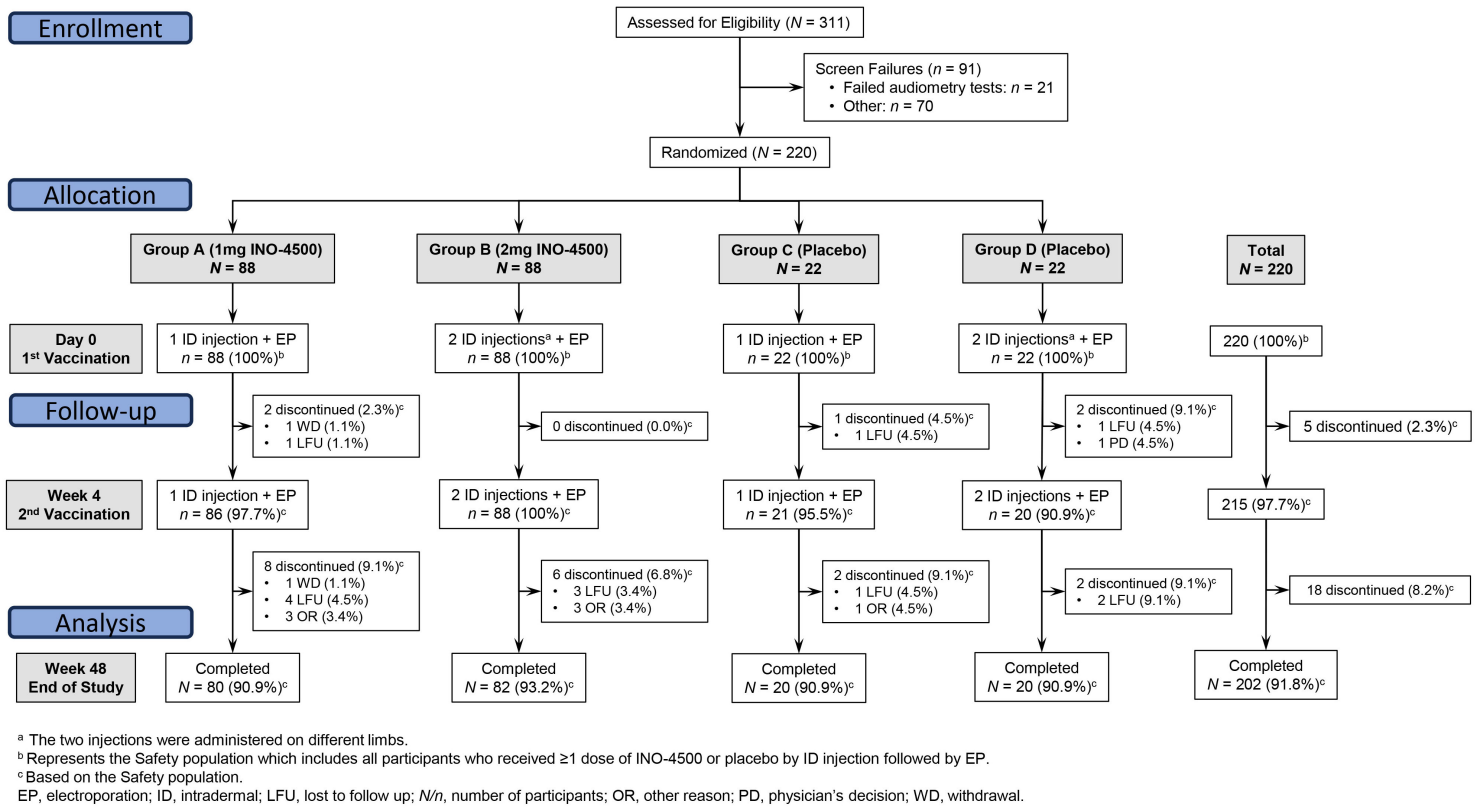
CONSORT diagram. LSV-002 was a Phase 1b, randomized, blinded, placebo-controlled trial enrolling participants at a ratio of 4:1 to receive a two-dose series of either INO-4500 or placebo. Eligibility was assessed for 311 subjects, and 220 participants were enrolled across four groups. Intervention arms were Group A with a single intradermal (ID) injection of 0.1 mL (1 mg) of INO-4500 on Day 0 and on Week 4; and Group B, with two ID injections (on separate limbs) of 0.1 mL (total dose of 2 mg) of INO-4500 on Day 0 and on Week 4. Placebo arms were Group C, with a single ID injection of 0.1 mL of saline sodium citrate (SSC) on Day 0 and on Week 4; and Group D, with two ID injections (on separate limbs) of 0.1 mL (total dose of 0.2 mL) of SSC on Day 0 and on Week 4. ID administration of INO-4500 or placebo was followed by electroporation (EP).

Potential participants were consented and screened. Participants meeting eligibility criteria were randomized in a blinded fashion to one of four groups across the two dose regimens, either receiving active investigational product (INO-4500; 176 participants) or placebo (saline sodium citrate [SSC] buffer solution; 44 participants) (**Figure 1**). Blinding of INO-4500 and placebo was in place for the participants, sponsor, and all clinical site staff, except for the site pharmacy staff who were not involved in any post-vaccination assessments. Group A received a single 1 mg INO-4500 (0.1 ml of a 10 mg/ml solution) ID injection at Day 0 and Week 4 (± five days), totaling 2 mg, while Group B received two 1 mg INO-4500 (0.1 ml of a 10 mg/ml solution) ID injections on different limbs at the same timepoints, totaling 4 mg. Group C received a single ID injection of 0.1 ml SSC (placebo) at Day 0 and Week 4 (± five days), while Group D received two ID injections of 0.1 ml SSC (placebo) on different limbs at the same time points. All ID injections were followed by EP using the CELLECTRA™ 2000 ID device (Inovio Pharmaceuticals) delivering four pulses of 52 milliseconds and 0.2 amps. For all groups, the first dose of INO-4500 or placebo dose occurred at Day 0, within 60 days of screening.

### Objectives and endpoints

The primary objectives of this clinical trial were to evaluate the tolerability and safety, as well as humoral and cellular immunogenicity of INO-4500 administered by ID injection followed by EP in healthy adult participants. For the safety evaluation, the incidence, severity, and relationship of adverse events (AEs) to the study intervention were collected.

#### Safety evaluations

Participants were observed for immediate reactions to the study treatment for 30 minutes after each vaccination (Day 0 and Week 4) and asked to attend the study clinic on Days 2, 14 (Week 2), 28 (Week 4), 42 (Week 6), 84 (Week 12), 168 (Week 24) and 336 (Week 48) (end of study assessment). Physical examinations were performed at all visits except Week 12 and Week 24. Medical/clinical assessment, collection of whole blood and serum for immunology assessment, and safety laboratory analyses were performed at each visit except Day 2. Solicited local (injection site) and systemic AEs were collected via participant diary cards on the evening of the day of vaccinations (Day 0 and Week 4) and seven subsequent days following each vaccination. The injection site reactions were graded as mild, moderate or severe – Grades 1, 2 and 3 respectively – in accordance with the “Toxicity Grading Scale for Healthy Adult and Adolescent Volunteers Enrolled in Preventive Vaccine Clinical Trials” (31).

Since hearing loss (HL) has been documented in LF survivors (32, 33), hearing assessments were conducted at screening, prior to second doses at Week 4 and at the final study visit at Week 48. Pure-tone audiometry was used to evaluate whether hearing was within normal-to-mild limits by evaluating hearing capabilities at certain frequencies: 500, 1000, 2000, 4000, 6000, and 8000 Hz. The hearing assessment included the hearing level (dB HL) at the measured frequencies. Degree of HL was defined as 0–15 dB: within normal limits; 16–25 dB: slight HL, 26–40 dB: mild HL; 41–55 dB: moderate HL; 56–70 dB: moderately severe HL; 71–90 dB: severe HL; 91+ dB: profound HL. Treatment-related attributable hearing loss measured as a change from baseline was reported as follows: AE, hearing loss of >30 dB in at least a single frequency and a change from one “Degree of Hearing Loss” category to another level, adverse event of special interest (AESI) as a hearing loss of >30 dB in three consecutive frequencies, and serious adverse event (SAE) as a hearing loss of ≥71 dB in at least one frequency (34).

AEs, including SAEs, treatment-emergent AEs (TEAEs), AESIs, as well as vital signs were evaluated throughout the study. The principal investigator (PI) assessed and graded clinical AEs or SAEs (based on discussions with participants) in accordance with the “Toxicity Grading Scale for Healthy Adult and Adolescent Volunteers Enrolled in Preventive Vaccine Clinical Trials” (31). AESIs relevant to development of a LF vaccine that were monitored for during the study included, but were not limited to, sensorineural hearing loss, encephalitis, and thrombocytopenia. No significant safety findings were noted to cause a safety pause to be requested by the Data Safety Monitoring Board.

#### Immunogenicity assessments

Whole blood and serum were collected for immunogenicity assessments prior to the first dose (at screening and/or Day 0), and during the study. Determination of analysis of collected samples for immunological endpoints was determined on an ongoing basis throughout the study.

Humoral immune responses were evaluated using a standard binding enzyme-linked immunosorbent assay (ELISA) developed at Inovio to measure levels of total IgG antibodies specific for glycoprotein (GP) antigen in serum isolated from whole blood samples drawn on Day 0, Week 6, and Week 12. This GP antigen was from lineage IV (Recombinant LASV L-IV Prefusion Glycoprotein, Zalgen Labs), one of the three most prevalent lineages of LASV in Sierra Leone, Guinea, and Liberia (35). Post-vaccination end point IgG titers were reported as the dilution of sera at which the average optical density (OD) of triplicate wells was greater than the assay cutoff and average OD of the negative control, then converted to concentration (IU/mL) using the First WHO International Standard for anti-Lassa fever virus antibodies (NIBSC code: 20/202). The assay limit of detection (LOD) was 9.065 IU/mL. Seroconversion rate was defined as a two-fold rise over baseline (Day 0) values.

Cellular immune responses were assessed using an interferon-gamma (IFN-γ) Enzyme-Linked ImmunoSpot (ELISpot) assay performed on peripheral blood mononuclear cells (PBMCs) isolated from whole blood collected at Day 0, and Weeks 2, 4, 6, 12, 24 and 48. The number of antigen-specific IFN-γ secreting cells was determined in response to stimulation with two pools (GP1, GP2) of 15-mer peptides overlapping by 9 amino acids spanning the LASV GP precursor. Phorbol 12-myristate 13-acetate (PMA) and ionomycin (a calcium ionophore) were used in combination to stimulate cells as a positive control, while cells with no stimulant were used as negative controls for all assays. The assay LOD was 11 spot forming units (SFU)/10^6^ PBMCs. ELISpot data were collected across two runs to support informal interim (Dataset 1) and long-term follow-up (Dataset 2) analyses. For the first analytical run (Dataset 1), samples collected at baseline (Day 0) and Weeks 6, 12, and 24 were analyzed. For the second analytical run (Dataset 2), samples collected at baseline and Weeks 2, 4, 24, and 48 were analyzed. Data were analyzed for Datasets 1 and 2 either individually or in combination, the latter using baseline subtraction. For each participant, a response threshold was calculated based on the mean SFU/10^6^ PBMCs plus two standard deviations of triplicate at baseline (Day 0) plus assay limit of quantitation (20 SFU/10^6^ PBMCs). Responder criterion was defined as any post-treatment value that exceeded the calculated response threshold.

### Statistics

The safety analysis included all participants in the Safety population, who received at least one dose of INO-4500 or placebo administered by ID injection. TEAEs were defined for this trial as any AEs/SAEs that occurred on or after Day 0. All TEAEs and serious TEAEs were summarized by frequency and percentage. Laboratory response variables were descriptively summarized by time point and as changes from baseline. As an early clinical phase study, the safety evaluation focused on identifying safety signals or concerns of clinical relevance rather than potential statistical differences. Thus, no formal statistical testing was performed.

All primary immunogenicity analyses were conducted on participants in the modified intent-to-treat (mITT) population. Similar to the Safety population, the mITT population included all participants who received at least one dose of INO-4500 or placebo. Binding antibody levels were analyzed by dose regimen up to Week 12 using geometric mean titers with interquartile range or fold rise with associated 95% confidence intervals. Antigen-specific cellular immune responses above baseline were analyzed by dose regimen up to Week 48 as SFUs per 10^6^ PBMCs with interquartile range or medians with 95% confidence intervals. Peak cellular response taken from any timepoint for each participant was used to calculate the mean peak SFU within each group. Percentage of participants with seroconversion (i.e., positive titer) or cellular response were analyzed within each study group. Statistical comparisons were performed within each study group between study weeks using the Wilcoxon signed-rank test and between groups at each study week using the Kruskal-Wallis test. The false discovery rate for multiple comparisons was controlled using the Benjamini-Hochberg adjustment and *p*_adj_-values are reported. Statistical tests were performed using GraphPad Prism version 10.5.0.

## Results

### Participant demographics and baseline characteristics

A total of 311 participants were screened for eligibility, with 220 participants subsequently enrolled and randomized at a single clinical trial site in Accra, Ghana, between December 2020 and September 2021 (**Figure 1**). The 220 study participants were on average 22 years old (range, 18-43), 86% were males, and the mean Body Mass Index was 21.8 kg/m^2^ (range, 16.6-35.4), very similar across study groups (**Table 1**).

**Table 1:** Demographic characterization of the LSV-002 study population.

Parameter	INO-4500 arms (*N* = 176)		Placebo arms (*N* = 44)		Total (*N* = 220)
	Group A (*N* = 88)	Group B (*N* = 88)	Group C (*N* = 22)	Group D (*N* = 22)	
Age in Years					
Mean (SD)	22.3 (4.5)	22.9 (4.22)	22.4 (2.5)	24.4 (5.8)	22.8 (4.4)
Median	21.5	22.0	22	23	22.0
Range	18-43	18-40	18-29	19-42	18-43
Sex, n (%)					
Females	16 (18.2)	7 (8.0)	4 (18.2)	3 (13.6)	30 (13.6)
Males	72 (81.8)	81 (92.0)	18 (81.8)	19 (86.4)	190 (86.4)
Race, n (%)					
African	88 (100.0)	88 (100.0)	22 (100.0)	22 (100.0)	220 (100.0)
Height in Centimeters					
Mean (SD)	172.4 (7.3)	172.8 (7.1)	173.9 (8.4)	171.8 (5.0)	172.7 (7.1)
Range	155.0-190.6	150.2-188.2	157.2-187.5	160.0-181.2	150.2-190.6
Weight in Kilograms					
Mean (SD)	66.4 (11.1)	67.8 (11.9)	68.6 (13.3)	66.7 (8.5)	67.2 (11.4)
Range	48.0-100.0	49.0-105.0	46.0-98.0	49.0-87.0	46.0-105.0
Body Mass Index in Kilograms/meter^2^					
Mean (SD)	22.4 (3.7)	22.7 (3.6)	22.6 (3.7)	22.6 (3.0)	22.6 (3.5)
Range	16.6-35.4	16.8-32.1	16.7-32.0	17.6-29.2	16.6-35.4

*N/n*, number of participants; SD, standard deviation.

LSV-002 Study had four groups. Intervention arms were Group A with a single intradermal (ID) injection of 0.1 mL (1 mg) of INO-4500 on Day 0 and on Week 4; and Group B, with two ID injections (on separate limbs) of 0.1 mL (total dose of 2 mg) of INO-4500 on Day 0 and on Week 4. Placebo arms were Group C, with a single ID injection of 0.1 mL saline sodium citrate (SSC) buffer solution on Day 0 and on Week 4; and Group D, with two ID injections (on separate limbs) of 0.1 mL (total dose of 0.2 mL) of SSC on Day 0 and on Week 4. ID administration of INO-4500 or placebo was followed by electroporation (EP). In table, values are rounded up to the first decimal point.

### Study procedure

Of the 220 randomized participants, 88 received 1 mg INO-4500 (Group A), 88 received 2 mg INO-4500 (Group B), 22 received placebo in each of two groups (Groups C and D) (**Figure 1**). All participants received at least one dose of INO-4500 or placebo followed by EP. Overall, most participants received all planned study doses (215 of 220, 97.7%) and 97.9% (646/660) of planned injections were administered. Of the eighteen participants who did not complete all study visits, ten were lost to follow-up, one participant withdrew, and seven did not complete the study for other reasons.

### Safety assessments

A summary of AEs is provided in **Table 2**. Of the 220 participants in the study, 197 (89.5%) reported 906 AEs. All but seven AEs (899 AEs) were TEAEs. Among participants, 195 (88.6%), and 29 (13.2%) experienced at least one Grade 1 and Grade 2 TEAE. A single Grade 3 and a single Grade 4 TEAE was experienced by 6 (2.7%) and 1 (0.5%) participants, respectively. Eight participants (3.6%) experienced one SAE each. There were no SAEs preceding initiation of treatment. None of the AEs led to discontinuation or death.

**Table 2:** Summary of adverse events in the LSV-002 study safety population.

Parameter	INO-4500 arms				Placebo arms				Total (*N* = 220)	
	Group A (*N* = 88)		Group B (*N* = 88)		Group C (*N* = 22)		Group D (*N* = 22)			
	Participants (%)	Number events	Participants (%)	Number events	Participants (%)	Number events	Participants (%)	Number events	Participants (%)	Number events
Participants with ≥1 adverse events (AE)	80 (90.9)	375	82 (93.2)	382	16 (72.7)	66	19 (86.4)	83	197 (89.5)	906
Participants with ≥1 treatment-emergent AE (TEAE)	80 (90.9)	375	82 (93.2)	379	16 (72.7)	64	19 (86.4)	81	197 (89.5)	899
Participants with at Least One Pre-treatment AE	0 (0.0)	0	2 (2.3)	3	1 (4.5)	2	2 (9.1)	2	5 (2.3)	7
Participants with ≥1 treatment-emergent SAE (TESAE)	3 (3.4)	3	4 (4.5)	4	0 (0.0)	0	1 (4.5)	1	8 (3.6)	8
Participants with ≥1 Grade 1 TEAE	79 (89.8)	352	81 (92.0)	357	16 (72.7)	63	19 (86.4)	78	195 (88.6)	850
Participants with ≥1 Grade 2 TEAE	12 (13.6)	20	14 (15.9)	19	1 (4.5)	1	2 (9.1)	2	29 (13.2)	42
Participants with ≥1 Grade 3 TEAE	2 (2.3)	2	3 (3.4)	3	0	0	1 (4.5)	1	6 (2.7)	6
Participants with ≥1 Grade 4 TEAE	1 (1.1)	1	0 (0.0)	0	0 (0.0)	0	0 (0.0)	0	1 (0.5)	1
Participants with ≥1 TEAE leading to death	0 (0.0)	0	0 (0.0)	0	0 (0.0)	0	0 (0.0)	0	0 (0.0)	0
Participants with ≥1 TEAE leading to treatment discontinuation	0 (0.0)	0	0 (0.0)	0	0 (0.0)	0	0 (0.0)	0	0 (0.0)	0
Participants with ≥1 treatment-related TEAE (TR-TEAE)	70 (79.5)	277	73 (83.0)	295	15 (68.2)	50	17 (77.3)	63	175 (79.5)	685
Participants with ≥1 serious TR-TEAE	0 (0.0)	0	0 (0.0)	0	0 (0.0)	0	0 (0.0)	0	0 (0.0)	0

*N*, number of participants; AE, adverse event; TEAE, treatment-emergent adverse event; SAE, serious adverse event; TESAE, treatment-emergent serious adverse event; AESI, adverse event of special interest; TR-TEAE, treatment-related treatment-emergent adverse event.

LSV-002 Study had four groups. Intervention arms were Group A with a single intradermal (ID) injection of 0.1 mL (1 mg) of INO-4500 on Day 0 and on Week 4; and Group B, with two ID injections (on separate limbs) of 0.1 ml (total dose of 2 mg) of INO-4500 on Day 0 and on Week 4. Placebo arms were Group C, with a single ID injection of 0.1 mL of saline sodium citrate (SSC) buffer solution on Day 0 and on Week 4; and Group D, with two ID injections (on separate limbs) of 0.1 mL (total dose of 0.2 mL) of SSC on Day 0 and on Week 4. ID administration of INO-4500 or placebo was followed by electroporation (EP). Adverse events (AE) were coded using the Medical Dictionary for Regulatory Activities (MedDRA) version 25.0. Grading scale is based on the Common Terminology Criteria for Adverse Events (CTCAE) v5.0. AEs includes all treatment-emergent AEs for subjects. At each level of subject summarization, a participant is counted once if the participant reported one or more events. In table, values were rounded up to the first decimal point.

Three participants experienced a single AESI each: thrombocytopenia in a subject in INO-4500 Group A and placebo Group C, and gingival bleeding in a subject in INO-4500 Group B. All three AESIs were assessed as non-serious and not related to clinical trial treatment. No other AESIs including cases of attributable hearing loss, either self-reported or detected by audiometry, were observed during the trial (**Supplementary Table S1**).

Of the seven Grade ≥3 TEAE, six occurred in participants receiving INO-4500 and consisted of peptic ulcer, gastroenteritis (two), elevated aspartate aminotransferase, headache, and seizure. The only Grade 4 event was a headache. A case of malaria was the one TEAE Grade 3 occurring in a participant receiving placebo (**Table 3**).

**Table 3:** Treatment-emergent adverse events by preferred term including high grade, seriousness, and relation to treatment in LSV-002 study safety population.

Adverse events	INO-4500 arms		Placebo arms		Total (*N* = 220)
	Group A (*N* = 88)	Group B (*N* = 88)	Group C (*N* = 22)	Group D (*N* = 22)	
	Participants (%)	Participants (%)	Participants (%)	Participants (%)	Participants (%)
Grade ≥3 Treatment-Emergent Adverse Events (TEAE)					
Number of Grade ≥3 TEAE*	3	3	0	1	7
Participants with ≥1 Grade ≥3 TEAE	3 (3.4)	3 (3.4)	0 (0.0)	1 (4.5)	7 (3.2)
Preferred Term					
Peptic ulcer	0 (0.0)	1 (1.1)	0 (0.0)	0 (0.0)	1 (0.5)
Gastroenteritis	1 (1.1)	1 (1.1)	0 (0.0)	0 (0.0)	2 (0.9)
Malaria	0 (0.0)	0 (0.0)	0 (0.0)	1 (4.5)	1 (0.5)
Aspartate aminotransferase increased	1 (1.1)	0 (0.0)	0 (0.0)	0 (0.0)	1 (0.5)
Headache	1 (1.1)	0 (0.0)	0 (0.0)	0 (0.0)	1 (0.5)
Seizure	0 (0.0)	1 (1.1)	0 (0.0)	0 (0.0)	1 (0.5)
Treatment-Emergent Serious Adverse Events (TESAE)					
Number of TESAE	3	4	0	1	8
Participants with ≥1 TESAEs	3 (3.4)	4 (4.5)	0 (0.0)	1 (4.5)	8 (3.6)
Preferred Term					
Peptic ulcer	0 (0.0)	1 (1.1)	0 (0.0)	0 (0.0)	1 (0.5)
Gastroenteritis	1 (1.1)	1 (1.1)	0 (0.0)	0 (0.0)	2 (0.9)
Malaria	1 (1.1)	1 (1.1)	0 (0.0)	1 (4.5)	3 (1.4)
Fibroma	1 (1.1)	0 (0.0)	0 (0.0)	0 (0.0)	1 (0.5)
Seizure	0 (0.0)	1 (1.1)	0 (0.0)	0 (0.0)	1 (0.5)
Treatment-Related Treatment-Emergent Adverse Events (TR-TEAE)					
Number of TR-TEAE	277	295	50	63	685
Participants with ≥1 TR-TEAE	70 (79.5)	73 (80)	15 (68.2)	17 (77.3)	175 (79.5)
Preferred Term with incidence rate ≥5%					
Injection site pain	57 (64.8)	62 (70.5)	13 (59.1)	15 (68.2)	147 (66.8)
Injection site swelling	51 (58.0)	52 (59.1)	7 (31.8)	10 (45.5)	120 (54.5)
Injection site bruising	50 (56.8)	48 (54.5)	7 (31.8)	11 (50.0)	116 (52.7)
Injection site erythema	10 (11.4)	13 (14.8)	0 (0.0)	0 (0.0)	23 (10.5)
Injection site pruritus	8 (9.1)	11 (12.5)	3 (13.6)	1 (4.5)	23 (10.5)
Malaise	0 (0.0)	1 (1.1)	0 (0.0)	2 (9.1)	3 (1.4)
Pyrexia	2 (2.3)	3 (3.4)	1 (4.5)	2 (9.1)	8 (3.6)
Headache	8 (9.1)	2 (2.3)	1 (4.5)	4 (18.2)	15 (6.8)

*N*, number of participants; TEAE, treatment-emergent adverse event; TESAE, treatment-emergent serious adverse event; TR-TEAE, treatment-related treatment-emergent adverse event.

LSV-002 Study had four groups. Intervention arms were Group A with a single intradermal (ID) injection of 0.1 mL (1 mg) of INO-4500 on Day 0 and on Week 4; and Group B, with two ID injections (on separate limbs) of 0.1 mL (total dose of 2 mg) of INO-4500 on Day 0 and on Week 4. Placebo arms were Group C, with a single ID injection of 0.1 mL of saline sodium citrate (SSC) buffer solution on Day 0 and on Week 4; and Group D, with two ID injections (on separate limbs) of 0.1 mL (total dose of 0.2 mL) of SSC on Day 0 and on Week 4. ID administration of INO-4500 or placebo was followed by electroporation (EP). Adverse events (AE) were coded using the Medical Dictionary for Regulatory Activities (MedDRA) version 25.0. Grading scale is based on the Common Terminology Criteria for Adverse Events (CTCAE) v5.0. In table, values were rounded up to the first decimal point.

*Of the 7 TEAE Grade ≥3, one was a Grade 4, (headache) occurring in Group A.

Of the eight serious TEAEs, seven occurred in participants receiving INO-4500, three in Group A and four in Group B, and included peptic ulcer, gastroenteritis (two), malaria (two), fibroma, and seizure. The self-reported single episode compatible with a seizure occurred 48 days post administration of the second dose of INO-4500 in the context of self-medication with an over-the-counter medicine available in Ghana that can cause seizures. Clinical and paraclinical repeated evaluations found no abnormalities, and the subject evolved favorably. A case of malaria was categorized as a serious TEAE in a participant receiving placebo. None of these TEAEs were considered related to the treatment and none led to study withdrawal (**Table 3**).

A total of 175 participants (79.5%) experienced a total of 685 TEAEs that were assessed as treatment-related by the investigators. The most frequently reported (≥5%) treatment-related TEAEs overall were localized to the injection site, including pain (66.8%), swelling (54.5%), bruising (52.7%), erythema (10.5%) and pruritus (10.5%). Three systemic treatment-related TEAE had an incidence ≥5% in at least one treatment group: malaise (9.1% in placebo Group D), pyrexia (9.1% in placebo Group D) and headache (9.1% and 18.2% in INO-4500 Group A and placebo Group D, respectively). **Table 3** details the incidence of treatment-related TEAE by treatment groups.

No clinically meaningful trends or abnormalities were observed in clinical laboratory measurements or physical examination findings throughout the study.

### Humoral immune responses

**Figure 2:**
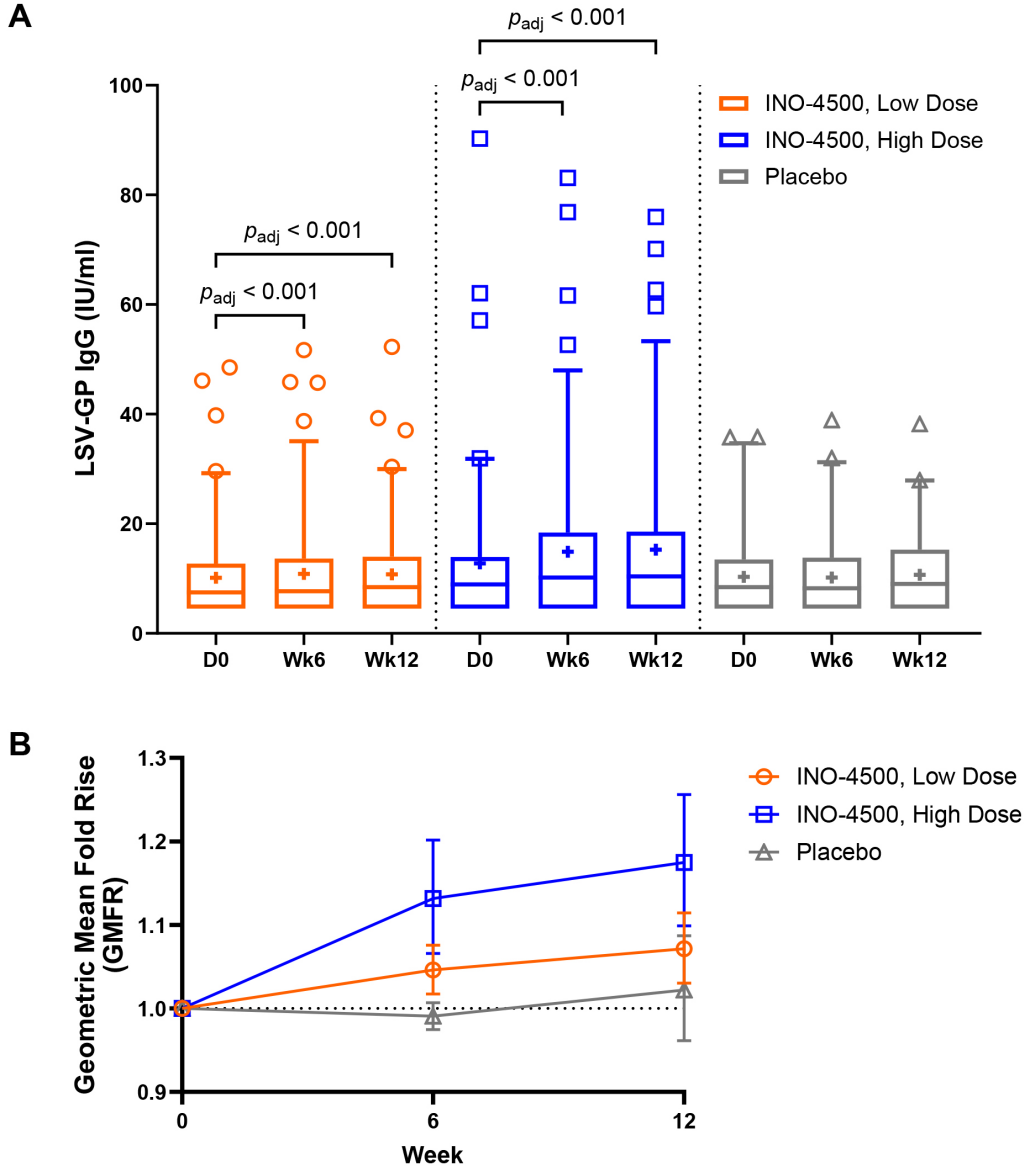
LASV GP-specific binding antibodies as measured by ELISA. LASV GP-specific binding antibodies are shown for each group. Group A (up to *n* = 85) participants received a total of 1 mg INO-4500 (low-dose) at each dosing visit, whereas Group B (up to *n* = 88) participants received 2 mg INO-4500 (high-dose). Placebo groups (Groups C and D; up to *n* = 42) are combined. **(A)** Box and whiskers extend from 25th to 75th and 5th to 95th percentiles, respectively, with outliers represented by open symbols. Line at the median; + at mean. *p*_adj_-values were calculated at each study week compared to Day 0 within each group using the Wilcoxon signed-rank test and between groups at each timepoint using the Kruskal-Wallis test; each test was followed by Benjamini-Hochberg adjustment. Only significant *p*_adj_-values displayed. **(B)** Geometric Mean Fold Rise (GMFR) of LASV GP-specific binding antibodies in each group represented by open symbols. Error bars show 95% CI. *p*_adj_-values calculated between groups at each post-vaccination timepoint using the Kruskal-Wallis test followed by Benjamini-Hochberg adjustment are displayed in **Table 5**. LASV, Lassa virus; GP, glycoprotein; ELISA, enzyme-linked immunosorbent assay; Ig, immunoglobulin; IU, International Unit; mL, milliliters; CI, confidence interval.

To assess the humoral immune responses induced by INO-4500, LASV GP binding ELISA was performed using sera collected from participants both before and after immunization. Participants treated with either low-dose (Group A, 1 mg) or high-dose (Group B, 2 mg) INO-4500 exhibited statistically significant increases in LASV GP-specific binding antibodies at Week 6 and Week 12 compared to Day 0 levels, while those who received placebo did not (**Figure 2A**, **Table 4**). Geometric mean fold rise (GMFR) analysis demonstrated a statistically significant increase in LASV GP-specific binding antibodies in both INO-4500 groups compared to placebo at Week 6 (low-dose, *p*_adj_ = 0.019; high-dose, *p*_adj_ *<*0.001) and Week 12 (low-dose, *p*_adj_ = 0.050; high-dose, *p*_adj_ *<*0.001), and the high-dose group also showed significantly greater responses than the low-dose group at both Week 6 (*p*_adj_ = 0.019) and Week 12 (*p*_adj_ = 0.024) (**Figure 2B**, **Table 5**). LASV GP-specific binding antibodies peaked at Week 12 in participants receiving either low- or high-dose INO-4500, with 3.6% and 9.3% responders, respectively, defined as two-fold rise over baseline (**Table 6**). While INO-4500 induced humoral immune responses that were not only detectable but also significantly increased, the magnitude of the antibody response was modest, resulting in few participants that met the threshold for seroreactivity.

**Table 4:** Geometric mean concentration (GMC) of LASV GP-specific binding antibodies as measured by ELISA in LSV-002 study.

Group	GMC, IU/mL (Min-Max)^a^		
	Day 0	Week 6	Week 12
INO-4500, Low-Dose^b^	8 (5–49)	8 (5–52)	8 (5-52)
INO-4500, High-Dose^b^	9 (5-90)	10 (5-83)	11 (5-76)
Placebo^c^	8 (5-36)	8 (5-39)	9 (5-38)

LASV, Lassa virus; GP, glycoprotein; GMC, Geometric Mean Concentration; IU, International Units; mL, milliliters; Min, minimum; Max, maximum.

a. Rounded to the nearest whole number.

b. 1 mg INO-4500 was injected ID followed by EP on one (Low-Dose, Group A) or two (High-Dose, Group B) different limbs at each dosing visit.

c. Placebo groups (Groups C and D) are combined.

**Table 5:** Statistical comparison between groups for geometric mean fold rise (GMFR) of LASV GP-specific binding antibodies as measured by ELISA in LSV-002 study.

Group	*p*_adj_-value^a^	
	Week 6	Week 12
INO-4500: Low- *vs*. High-Dose	0.019	0.024
INO-4500, Low-Dose *vs*. Placebo^b^	0.019	0.050
INO-4500, High-Dose *vs*. Placebo^b^	<0.001	<0.001

LASV, Lassa virus; GP, glycoprotein; ELISA, enzyme-linked immunosorbent assay.

1 mg INO-4500 was injected ID followed by EP on one (Low-Dose, Group A) or two (High-Dose, Group B) different limbs at each dosing visit.

a. *p* **_adj_**-values calculated between groups at each study week using the Kruskal-Wallis test followed by Benjamini-Hochberg adjustment.

b. Placebo groups (Groups C and D) are combined.

**Table 6:** Humoral immune responses to LASV GP as measured by ELISA in LSV-002 study.

Group	Response, %^a^ (*n*/*N*)		
	Week 6	Week 12	Any timepoint^b^
INO-4500, Low-Dose^c^	1.2 (1/85)	3.6 (3/84)	3.5 (3/85)
INO-4500, High-Dose^c^	5.7 (5/88)	9.3 (8/86)	9.1 (8/88)
Placebo^d^	0.0 (0/42)	2.4 (1/41)	2.4 (1/42)

LASV, Lassa virus; GP, glycoprotein; ELISA, enzyme-linked immunosorbent assay; %, percent; *n/N*, number of participants.

a. % rounded to the nearest tenth.

b. Best responses taken from any post-Day 0 timepoint.

c. 1 mg INO-4500 was injected ID followed by EP on one (Low-Dose, Group A) or two (High-Dose, Group B) different limbs at each dosing visit.

d. Placebo groups (Groups C and D) are combined.

### Cellular immune responses

**Figure 3:**
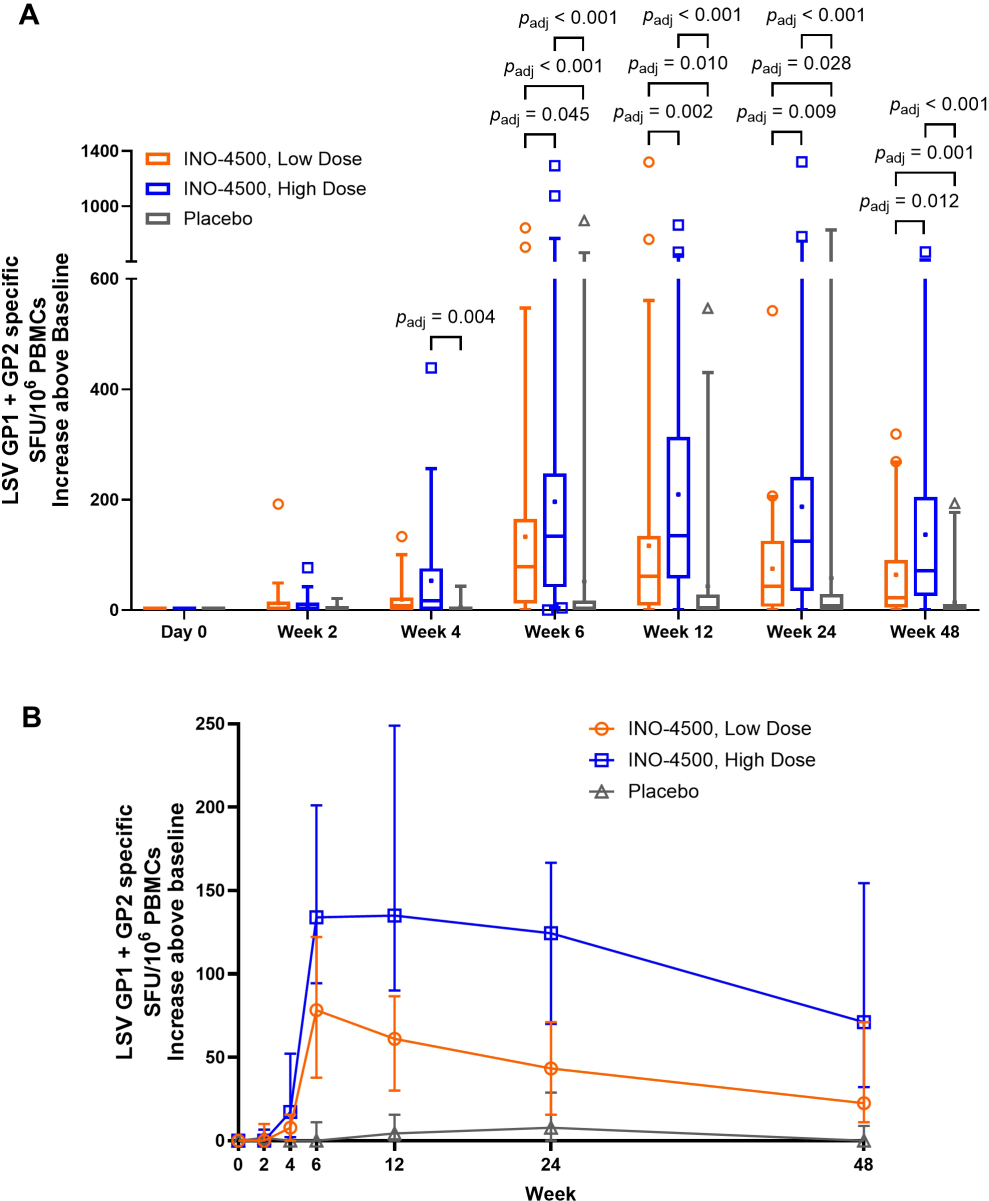
Cellular immune responses to LASV as measured by IFN-γ ELISpot. Baseline subtracted LASV-specific SFU per million PBMCs in response to total LASV (the sum of glycoprotein peptide pools GP1 and GP2) are shown for each group with Datasets 1 (interim analysis) and 2 (long-term follow-up analysis) combined. Placebo groups (Groups C and D) are combined. The larger of two magnitudes above baseline were excluded for six participants with duplicate Week 24 data. Group A, up to *n* = 59; Group B, up to *n* = 66; Placebo, up to *n* = 31. **(A)** Box and whiskers extend from 25th to 75th and 5th to 95th percentiles, respectively, with outliers represented by open symbols. Line at the median; + at mean. *p*_adj_-values were calculated at each study week between groups using the Kruskal-Wallis test followed by Benjamini-Hochberg adjustment. Only significant *p*_adj_-values displayed. **(B)** Medians with 95% CI are represented by open symbols for each group. LASV, Lassa virus; GP, glycoprotein; IFN-γ, interferon-gamma; ELISpot, Enzyme-linked immunosorbent spot; SFU, spot forming units; PBMCs, peripheral blood mononuclear cells; CI, confidence interval.

**Figure 4:**
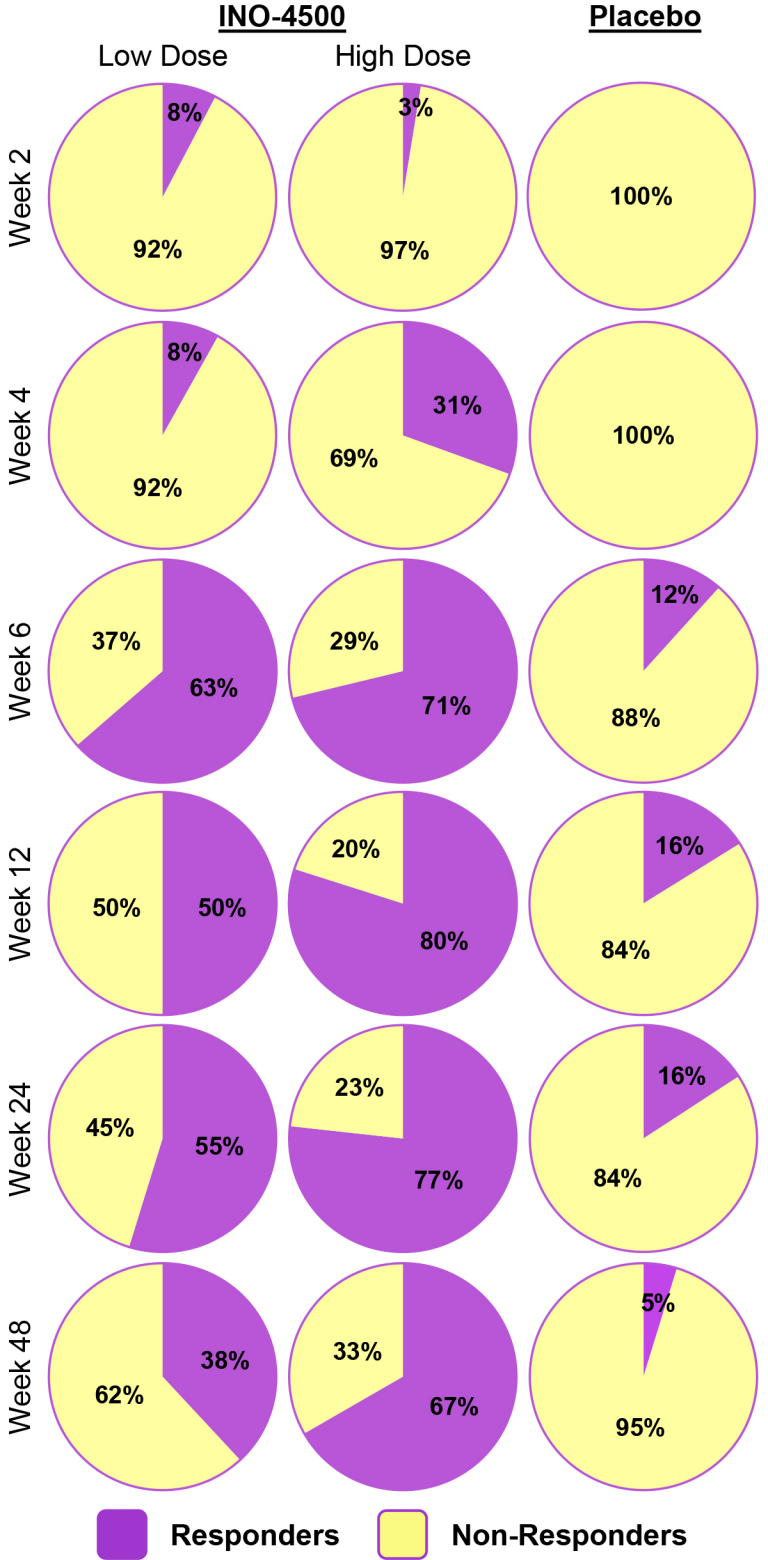
Responder status to total LASV GP as measured by IFN-γ ELISpot by group. Percent participants who displayed a cellular immune response to stimulation with total LASV GP (the sum of glycoprotein peptide pools GP1 and GP2) for each group at each timepoint with Datasets 1 (interim analysis) and 2 (long-term follow-up analysis) combined. Placebo groups (Groups C and D) are combined. Group A, up to *n* = 59; Group B, up to *n* = 66; Placebo, up to *n* = 31. Responder status was assigned to any of the six participants with duplicate Week 24 data only if each were considered responders for both Datasets 1 and 2. LASV, Lassa virus; GP, glycoprotein; IFN-γ, interferon-gamma; ELISpot, Enzyme-linked immunosorbent spot.

To evaluate INO-4500-induced cellular immune responses, an IFN-γ ELISpot assay was performed on PBMCs collected pre-vaccination and at multiple timepoints during the study. This assay detected and quantified IFN-γ secreting cells following stimulation with antigens (LASV GP-derived peptide pools GP1 and GP2). Participants who received either low- (Group A) or high-dose (Group B) INO-4500 exhibited increases in LASV GP-specific IFN-γ secreting T cells after vaccination (**Figure 3**, combined dataset; **Supplementary Figure S1** datasets 1 and 2). Additionally, both groups showed a statistically significant increase in LASV GP-specific T cell response at Weeks 4, 6, 12, 24, and 48 as compared to those who received placebo (**Figure 3A**). The high-dose INO-4500 group exhibited greater responses than the low-dose group from Week 6 through Week 48. T cell response magnitude peaked at Week 6 in the low-dose group and at 12 weeks in the high-dose group, with elevated responses sustained above the Day 0 responses through the end of the study at Week 48 (**Figure 3B**). Responder rates, defined by positive IFN-γ responses to GP1 and GP2, peaked with 63% at Week 6 for low-dose and 80% at Week 12 for high-dose recipients (**Figure 4**, combined dataset; **Supplementary Figure S2**, datasets 1 and 2). The 2 mg dose also resulted in a better response rate for participants than the 1 mg dose at Week 6 (71% versus 63%, respectively). Rates of T cell response at any timepoint were 75% and 86% for participants who received low- and high-dose INO-4500, respectively, compared to 26% for those who received placebo (**Table 7**, combined dataset; **Supplementary Table S2**, dataset 1; **Supplementary Table S3**, dataset 2). Participants from the high-dose INO-4500 group generated the highest mean peak responses to stimulation with total LASV GP (324 SFU/10^6^ PBMCs) followed by low-dose INO-4500 (198 SFU/10^6^ PBMCs) then placebo (103 SFU/10^6^ PBMCs) groups.

**Table 7:** Cellular immune responses to LASV GP at any timepoint as measured by IFN-γ ELISpot, combined datasets in LSV-002 study.

LASV pool	INO-4500, low-dose^a^		INO-4500, high-dose^a^		Placebo^b^	
	Response, % (*n*/*N*)	Mean peak SFU	Response, % (*n*/*N*)	Mean peak SFU	Response, % (*n*/*N*)	Mean peak SFU
GP1	52.5 (31/59)	79.1	59.1 (39/66)	106.1	25.8 (8/31)	50.8
GP2	69.5 (41/59)	123.5	84.8 (56/66)	221.3	19.4 (6/31)	52.9
Total	74.6 (44/59)	198.1	86.4 (57/66)	324.4	25.8 (8/31)	102.6

LASV, Lassa virus; GP, glycoprotein; IFN-γ, interferon-gamma; ELISpot, enzyme-linked immunosorbent spot assay; %, percent; *n*/*N*, number of participants; SFU, spot forming units.

All data for % response and mean peak were rounded to the nearest tenth.

a. 1 mg INO-4500 was injected ID followed by EP on one (Low-Dose, Group A) or two (High-Dose, Group B) different limbs at each dosing visit.

b. Placebo groups (Groups C and D) are combined.

## Discussion

Lassa fever remains a persistent public health threat in West Africa, yet no licensed vaccine is currently available. Although research began in the 1970s, progress has been limited by the extensive genetic diversity of LASV strains and insufficient surveillance data (12, 36). In more recent years, the disease has gained global attention and was added to the WHO Research and Development Roadmap (9). Since 2015, 34 vaccine candidates have been developed, and four are currently in clinical trials: INO-4500, rVSVΔG-LASV-GPC, MV-LASV, and EBS-LASV (12, 36, 37). This includes a CEPI-supported Phase II clinical trial ongoing in West Africa to evaluate a recombinant vesicular stomatitis virus (rVSV) vector vaccine, rVSVΔG-LASV-GPC (10, 14). INO-4500 is notably the only DNA-based vaccine investigated in clinical trials (37).

One of the scenarios of the WHO’s target product profile (TPP) for development of a LASV vaccine focuses on non-emergency, preventive vaccine campaigns for those living in endemic areas and individuals such as healthcare workers at high risk of exposure (38). This approach is further supported by a recent epidemiological modeling study supported by CEPI and conducted by the University of Liverpool and the University of Oxford. The authors analyzed the projected impact of six LASV vaccination scenarios over a 10-year period. The analysis found that preventative vaccination strategies targeting endemic areas were significantly more effective than reactive vaccination based on estimated rates of infections, hospitalizations, deaths, disability-adjusted life years (DALYs), and societal expenditures. Of the estimated 2.7 million LASV infections occurring annually in West Africa, causing a burden of approximately 200,000 DALYs, population-wide preventative vaccination in a non-emergency setting could potentially save $20.1 million in DALY losses and $128.8 million in societal costs – ten times more than a reactive outbreak response (4). Further, a 2023 survey of eight West African-based LF experts also recommend prioritizing preventative vaccination of endemic areas due to the unclear cost-benefit ratio of reactive campaigns (39).

The Phase 1b study reported here provides important evidence supporting the safety, tolerability and immunogenicity of INO-4500, a synthetic DNA vaccine encoding for LASV GPC, which was administered by ID injection followed by EP. Building on the encouraging results of an US-based Phase 1a trial (NCT03805984), in which we observed no SAEs or AESIs, the current LSV-002 study provides further evidence that INO-4500 administered ID and followed by EP exhibited a well-tolerated safety profile in a target population of healthy Ghanaian adults. The vaccine produced no Grade 3 or higher treatment-related TEAEs, and 88.6% of all TEAEs were Grade 1. Notably, no cases of hearing loss or sensorineural impairment, key concerns for LASV infection, were observed following INO-4500 vaccination in this study. Although sensorineural hearing loss occurs in approximately 30% of LF survivors (32, 33), evidence to date from both clinical trials and preclinical studies evaluating LF vaccines, including one evaluating INO-4500 in an LASV challenge study involving NHPs, has revealed no reports of hearing loss post-LASV vaccination (15, 33, 40).

Our results further support the clinical trial evidence regarding the favorable safety profile of the DNA vaccine platform. Across multiple studies utilizing this technology, the reported AEs were mild to moderate, with no evidence to date that they cause severe or persistent systemic AEs (41–46). Furthermore, the DNA vaccine platform has been shown to have a lower risk of local and systemic AEs following immunization as compared to alternative vaccine modalities (inactivated vaccine, mRNA, viral-based vector, and protein subunit vaccines), as reported in a meta-analysis by Kouhpayeh, et al. (47).

INO-4500 elicited humoral and cellular immune responses that were statistically significant over placebo. While both INO-4500 groups exhibited significant increases in LASV GP-specific binding antibodies at Weeks 6 and 12 compared to placebo, with the high-dose group eliciting the strongest responses, humoral response levels and seroconversion rate remained low (<10%) through Week 12. Negligible IgG and IgM antibody responses have been observed in viremic patients during acute LASV infection (48, 49), which may explain, in part, why low rates of seroconversion were observed after INO-4500 administration during the present study. Moreover, structural properties, such as the glycan shield, obscure the glycoprotein subunits of the LASV GPC (49) and the presence of irrelevant LASV GPC conformations (50) have been reported to mask key regions of the GPC, making it harder for antibodies, including neutralizing, to recognize and bind to their target. In fact, both LASV infection and vaccination have resulted in delayed and/or weak neutralizing antibody responses (49). For these reasons, neutralizing antibody responses were not assessed at the early timepoints indicated in the current study. However, characterization at both early and later timepoints may have offered additional insights into the humoral immune responses to vaccination with INO-4500.

Cellular immune responses were collected across two analytical runs to support informal interim and long-term follow-up analyses. When analyzed individually, responses from these two datasets differed in range and magnitude across time points, perhaps due in part to the range in number of participants tested at different timepoints across both datasets; however, overall trends were similar when analyzed in combination. INO-4500 induced T cell responses in both groups, with the high-dose group eliciting the strongest mean peak response followed by the low-dose group, which was two-fold greater than placebo. 71% and 80% of participants from the high-dose group responded at Week 6 and Week 12, respectively. Notably, T cell responses were durable and remained elevated above baseline through Week 48 in both INO-4500 groups, underscoring the vaccine’s lasting immunogenicity. Robust CD4+ and CD8+ T cell responses have been observed during and following LASV infection in LF survivors and are likely important for control and clearance (19, 51, 52). While cellular immune responses were assessed by ELISpot in the current study, deeper characterization of LASV-specific CD4+ and CD8+ T cell responses may have offered additional insights into the cellular immune responses to vaccination with INO-4500.

Although the 2-dose vaccine regimen examined in this study demonstrated an adequate and durable cellular immune response, the low humoral response reported may be seen as a limit for the use of INO-4500 as a preventative measure. It is worth noticing that INO-4500 vaccinated NHPs exposed to a lethal dose of LASV Josiah strain a year later survived while presenting low to no humoral response at the initiation of the challenge (15). Nevertheless, based on preclinical studies in NHPs with INO-4500 in which 2- and 3-dose regimens were evaluated, seroconversion rates and magnitude in binding, and more importantly, in neutralizing antibody levels, increased to a greater degree with a 3-dose vaccination schedule (Andrade V et al. [manuscript in preparation]). Echoing the clinical developments of other DNA vaccines (43, 45, 50), future clinical studies with INO-4500 could benefit from exploring a 3-dose regimen. Finally, while this study demonstrated durability of immune responses for 12 months, further research is needed to assess longer-term durability and achieve the minimum WHO TPP benchmark of three years.

The DNA medicine platform used as the basis for INO-4500 is uniquely positioned to prevent infectious diseases in non-emergency settings, and has shown promising results as an effective, versatile platform (43, 45, 46, 53, 54). Using this innovative technology, DNA vaccines can be rapidly designed using a common platform to express relevant antigens and readily integrated into large-scale manufacturing (55, 56). They are not constrained by ultra cold-chain requirements due to their thermostability at refrigerated temperatures, making them particularly well suited for deployment in endemic regions with limited infrastructure (57). Unlike viral vector platforms, DNA vaccines are conducive to repeated dosing without eliciting anti-vector immunity, a critical advantage for long-term immunization strategies (58, 59) which could require the use of booster doses, as is now being discussed for the prevention of Ebola Virus Disease (EVD) (60). These features align well with the logistical and public health demands of preparing for LASV outbreaks in endemic regions.

In conclusion, this study demonstrates the safety, tolerability, and immunogenicity of INO-4500 administered by ID injection and followed by EP. Continued development of INO-4500 would potentially provide access to a safe, well tolerated and effective vaccine for the prevention of Lassa fever in healthcare workers and those living in endemic areas ahead of outbreaks. Such a vaccine could substantially reduce the burden of LF in West Africa and mitigate the risk of broader regional or global spread, fulfilling an urgent global health priority.

## Data Availability

The datasets presented in this article are not readily available because restrictions may apply due to privacy reasons, regulatory submissions, and/or ongoing research projects. Requests to access the datasets should be directed to LH, Laurent.Humeau@inovio.com.
