# Supplementary material for: Safety, tolerability, and immunogenicity of INO-4500, a synthetic DNA-based vaccine against Lassa virus, in a phase 1b clinical trial in healthy Ghanaian adults
Source: Front Immunol. 2025 Oct 24;16:1658549. doi: 10.3389/fimmu.2025.1658549 (PMC12592798; doi:10.3389/fimmu.2025.1658549)
Supplement: Supplementary file 1 [file DataSheet1.pdf]

Supplementary Table 1. Summary of Hearing Assessments by Ear, Frequency and Visit in the LSV-002 Study Safety Population

Hearing Test Frequency – Ear	Frequency decibel hearing level (dbHL)*	INO-4500 Arms				Placebo Arms				Total (N=220)	
		Group A (N=88)		Group B (N=88)		Group C (N=22)		Group D (N=22)			
		Baseline (n=88)	Week 48 (n=83)	Baseline (n=88)	Week 48 (n=85)	Baseline (n=22)	Week 48 (n=21)	Baseline (n=22)	Week 48 (n=20)	Baseline (n=220)	Week 48 (n=209)
**500 Hz**											
Left	Mean (SD)	9.1 (5.06)	8.6 (5.65)	8.6 (4.72)	8.5 (5.28)	9.5 (4.61)	10.7 (5.07)	8.6 (5.81)	9.0 (5.53)	8.9 (4.94)	8.8 (5.43)
	Median	10.0	10.0	10.0	10.0	10.0	10.0	10.0	10.0	10.0	10.0
	Min, Max	0, 20	0, 25	0, 20	0, 25	0, 20	0, 20	0, 25	0, 15	0, 25	0, 25
Right	Mean (SD)	9.8 (5.11)	9.2 (5.09)	9.0 (4.66)	9.8 (5.48)	10.5 (4.86)	11.4 (4.78)	10.2 (4.75)	9.0 (5.98)	9.6 (4.87)	9.7 (5.32)
	Median	10.0	10.0	10.0	10.0	10.0	10.0	10.0	10.0	10.0	10.0
	Min, Max	0, 20	0, 20	0, 20	0, 25	5, 25	5, 20	5, 20	0, 20	0, 25	0, 25
**1000 Hz**											
Left	Mean (SD)	9.5 (5.01)	8.9 (5.47)	10.5 (4.55)	10.1 (6.02)	11.4 (5.16)	12.1 (5.61)	9.1 (4.79)	8.5 (5.64)	10.1 (4.84)	9.7 (5.78)
	Median	10.0	10.0	10.0	10.0	10.0	10.0	10.0	10.0	10.0	10.0
	Min, Max	0, 20	0, 20	0, 25	0, 25	0, 20	0, 25	0, 20	0, 20	0, 25	0, 25
Right	Mean (SD)	9.0 (5.13)	9.5 (4.98)	9.1 (4.12)	10.1 (5.70)	11.6 (5.21)	11.7 (5.77)	7.3 (5.50)	8.8 (5.59)	9.1 (4.87)	9.9 (5.43)
	Median	10.0	10.0	10.0	10.0	10.0	10.0	7.5	10.0	10.0	10.0
	Min, Max	0, 25	0, 20	0, 15	0, 25	0, 25	0, 20	0, 20	0, 20	0, 25	0, 25
**2000 Hz**											
Left	Mean (SD)	9.4 (5.51)	9.3 (6.09)	9.2 (5.82)	9.1 (6.32)	11.4 (5.60)	11.4 (6.35)	10.7 (6.23)	10.0 (7.25)	10.7 (6.23)	9.5 (6.32)
	Median	10.0	10.0	10.0	10.0	12.5	10.0	10.0	10.0	10.0	10.0
	Min, Max	0, 25	0, 30	0, 25	0, 30	0, 25	0, 25	0, 20	0, 25	0, 20	0, 30
Right	Mean (SD)	9.7 (5.13)	9.1 (6.16)	7.9 (5.45)	7.9 (6.04)	10.7 (7.29)	12.4 (6.25)	9.8 (6.07)	9.0 (7.36)	9.1 (5.65)	8.9 (6.32)
	Median	10.0	10.0	7.5	10.0	10.0	10.0	10.0	7.5	10.0	10.0
	Min, Max	0, 25	0, 25	0, 20	0, 20	0, 25	5, 30	0, 20	0, 20	0, 25	0, 30
**4000 Hz**											
Left	Mean (SD)	8.9 (5.80)	9.5 (6.47)	9.7 (6.20)	9.7 (7.13)	10.7 (5.41)	12.6 (7.00)	10 (4.08)	10.3 (6.58)	9.5 (5.77)	10.0 (6.82)
	Median	10.0	10.0	10.0	10.0	10.0	10.0	10.0	10.0	10.0	10.0
	Min, Max	0, 25	0, 25	0, 30	0, 25	0, 20	0, 30	5, 20	0, 25	0, 30	0, 30
Right	Mean (SD)	10.7 (6.57)	10.0 (7.16)	10.7 (5.88)	10.1 (6.70)	9.5 (5.22)	10.0 (5.48)	12.0 (5.70)	10.8 (7.99)	10.7 (6.08)	10.1 (6.86)
	Median	10.0	10.0	10.0	10.0	10.0	10.0	12.5	10.0	10.0	10.0
	Min, Max	0, 25	0, 25	0, 30	0, 30	0, 20	0, 20	5, 25	0, 30	0, 30	0, 30
**6000 Hz**											
Left	Mean (SD)	7.3 (7.46)	6.1 (6.36)	6.9 (7.01)	5.9 (6.51)	8.6 (5.81)	9.0 (6.64)	6.4 (6.40)	8.0 (8.01)	7.2 (7.01)	6.5 (6.65)
	Median	5.0	5.0	5.0	5.0	10.0	10.0	5.0	5.0	5.0	5.0
	Min, Max	0, 30	0, 25	0, 30	0, 30	0, 20	0, 20	0, 20	0, 25	0, 30	0, 30
Right	Mean (SD)	6.8 (5.83)	6.2 (6.32)	6.3 (5.83)	4.8 (5.92)	8.2 (6.08)	7.9 (6.81)	5.7 (4.95)	6.8 (6.93)	6.6 (5.97)	5.8 (6.31)
	Median	5.0	5.0	5.0	5.0	7.5	5.0	5.0	5.0	5.0	5.0
	Min, Max	0, 25	0, 25	0, 25	0, 25	0, 25	0, 20	0, 20	0, 25	0, 25	0, 25

8000 Hz											
Left	Mean (SD)	6.9 (6.27)	5.9 (6.77)	6.4 (7.27)	6.5 (7.72)	7.7 (6.50)	9.8 (7.50)	10.0 (8.45)	8.8 (8.87)	7.1 (6.97)	6.8 (7.50)
	Median	5.0	5.0	5.0	5.0	10.0	10.0	10.0	5.0	5.0	5.0
	Min, Max	0, 25	0, 25	0, 30	0, 30	0, 25	0, 25	0, 25	0, 25	0, 30	0, 30
Right	Mean (SD)	7.9 (7.30)	7.2 (8.20)	7.3 (6.86)	6.7 (7.09)	9.3 (7.91)	9.0 (7.85)	8.9 (8.30)	7.5 (8.03)	7.9 (7.27)	7.2 (7.69)
	Median	5.0	5.0	5.0	5.0	10.0	5.0	5.0	5.0	5.0	5.0
	Min, Max	0, 30	0, 30	0, 30	0, 35**	0, 25	0, 25	0, 25	0, 25	0, 30	0, 35

N/n, number of participants; dbHL, decibel hearing level; SD, standard deviation.

* Hearing assessments were performed using pure-tone audiometry at Screening, Week 4, and at Week 48 or any other study discontinuation visit. Hearing assessments included hearing level (dB HL) at the measured frequencies. Interpretation of results based on Liu Y, Ibekwe T, Kelso J, et al. Vaccine. 2020;38(30):4717-4731.; degree of hearing loss (HL) (pure tone threshold on hearing level audiogram) – 0-15 dB: within normal limits; 16-25 dB: slight HL; 26-40 dB: mild HL; 41-55 dB: moderate HL; 56-70 dB: moderately severe HL; 71-90 dB: severe HL; 91+ dB: profound HL.

** A single participant experienced a HL of slight degree (i.e., 35 dbHL) in a single ear, for a single frequency during a single visit.

LSV-002 Study had four arms. Intervention arms were Group A with a single intradermal (ID) injection of 0.1 mL (1 mg) of INO-4500 on Day 0 and on Week 4; and Group B, with two ID injections (on separate limbs) of 0.1 ml (total dose of 2 mg) of INO-4500 on Day 0 and on Week 4. Placebo arms were Group C, with a single ID injection of 0.1 mL of saline sodium citrate (SSC) buffer solution on Day 0 and on Week 4; and Group D, with two ID injections (on separate limbs) of 0.1 mL (total dose of 0.2 mL) of SSC buffer solution on Day 0 and on Week 4. ID administration of INO-4500 or placebo was followed by electroporation (EP).
