# Supplementary material for: Safety, tolerability, and immunogenicity of INO-4500, a synthetic DNA-based vaccine against Lassa virus, in a phase 1b clinical trial in healthy Ghanaian adults
Source: Front Immunol. 2025 Oct 24;16:1658549. doi: 10.3389/fimmu.2025.1658549 (PMC12592798; doi:10.3389/fimmu.2025.1658549)
Supplement: Supplementary file 2 [file DataSheet2.pdf]

Supplementary Table 2. Cellular Immune Responses to LASV GP at any timepoint as Measured by IFN-γ ELISpot, Dataset 1[a] in LSV-002 Study

LASV Pool	INO-4500, Low-Dose[b]		INO-4500, High-Dose[b]		Placebo[c]	
	Response, % (*n*/*N*)	Mean Peak SFU	Response, % (*n*/*N*)	Mean Peak SFU	Response, % (*n*/*N*)	Mean Peak SFU
GP1	49.2 (29/59)	73.9	54.5 (36/66)	103.6	22.6 (7/31)	46.6
GP2	67.8 (40/59)	122.8	84.8 (56/66)	210.5	16.1 (5/31)	46.0
Total	72.9 (43/59)	193.6	84.8 (56/66)	312.4	22.6 (7/31)	92.0

LASV, Lassa virus; GP, glycoprotein; IFN-γ, interferon-gamma; ELISpot, enzyme-linked immunosorbent spot assay; %, percent; *n/N*, number of participants; SFU, spot forming units.

All data for % response and mean peak were rounded to the nearest tenth.

a. Interim analysis from baseline, Weeks 6, 12, 24.

b. 1 mg INO-4500 was injected ID followed by EP on one (Low-Dose, Group A) or two (High-Dose, Group B) different limbs at each dosing visit.

c. Placebo groups (Groups C and D) are combined.
