# Supplementary material for: Safety, tolerability, and immunogenicity of INO-4500, a synthetic DNA-based vaccine against Lassa virus, in a phase 1b clinical trial in healthy Ghanaian adults
Source: Front Immunol. 2025 Oct 24;16:1658549. doi: 10.3389/fimmu.2025.1658549 (PMC12592798; doi:10.3389/fimmu.2025.1658549)
Supplement: Supplementary file 3 [file DataSheet3.pdf]

Supplementary Table 3. Cellular Immune Responses to LASV GP at any timepoint as Measured by IFN-γ ELISpot, Dataset 2[a] in LSV-002 Study

LASV Pool	INO-4500, Low-Dose[b]		INO-4500, High-Dose[b]		Placebo[c]	
	Response, % (*n*/*N*)	Mean Peak SFU	Response, % (*n*/*N*)	Mean Peak SFU	Response, %[c] (*n*/*N*)	Mean Peak SFU
GP1	26.7 (12/45)	35.6	40.5 (17/42)	47.9	4.2 (1/24)	18.0
GP2	40.0 (18/45)	49.2	61.9 (26/42)	104.4	4.2 (1/24)	15.5
Total	42.2 (19/45)	84.1	66.7 (28/42)	148.5	4.2 (1/24)	31.9

LASV, Lassa virus; GP, glycoprotein; IFN-γ, interferon-gamma; ELISpot, enzyme-linked immunosorbent spot assay; %, percent; *n/N*, number of participants; SFU, spot forming units.
All data for % response and mean peak were rounded to the nearest tenth.
a. Long-term follow-up analysis from baseline, Weeks 2, 4, 24, 48.
b. 1 mg INO-4500 was injected ID followed by EP on one (Low-Dose, Group A) or two (High-Dose, Group B) different limbs at each dosing visit.
c. Placebo groups (Groups C and D) are combined.
