# Supplementary figures and images for: Safety, tolerability, and immunogenicity of INO-4500, a synthetic DNA-based vaccine against Lassa virus, in a phase 1b clinical trial in healthy Ghanaian adults
Source: Front Immunol. 2025 Oct 24;16:1658549. doi: 10.3389/fimmu.2025.1658549 (PMC12592798; doi:10.3389/fimmu.2025.1658549)

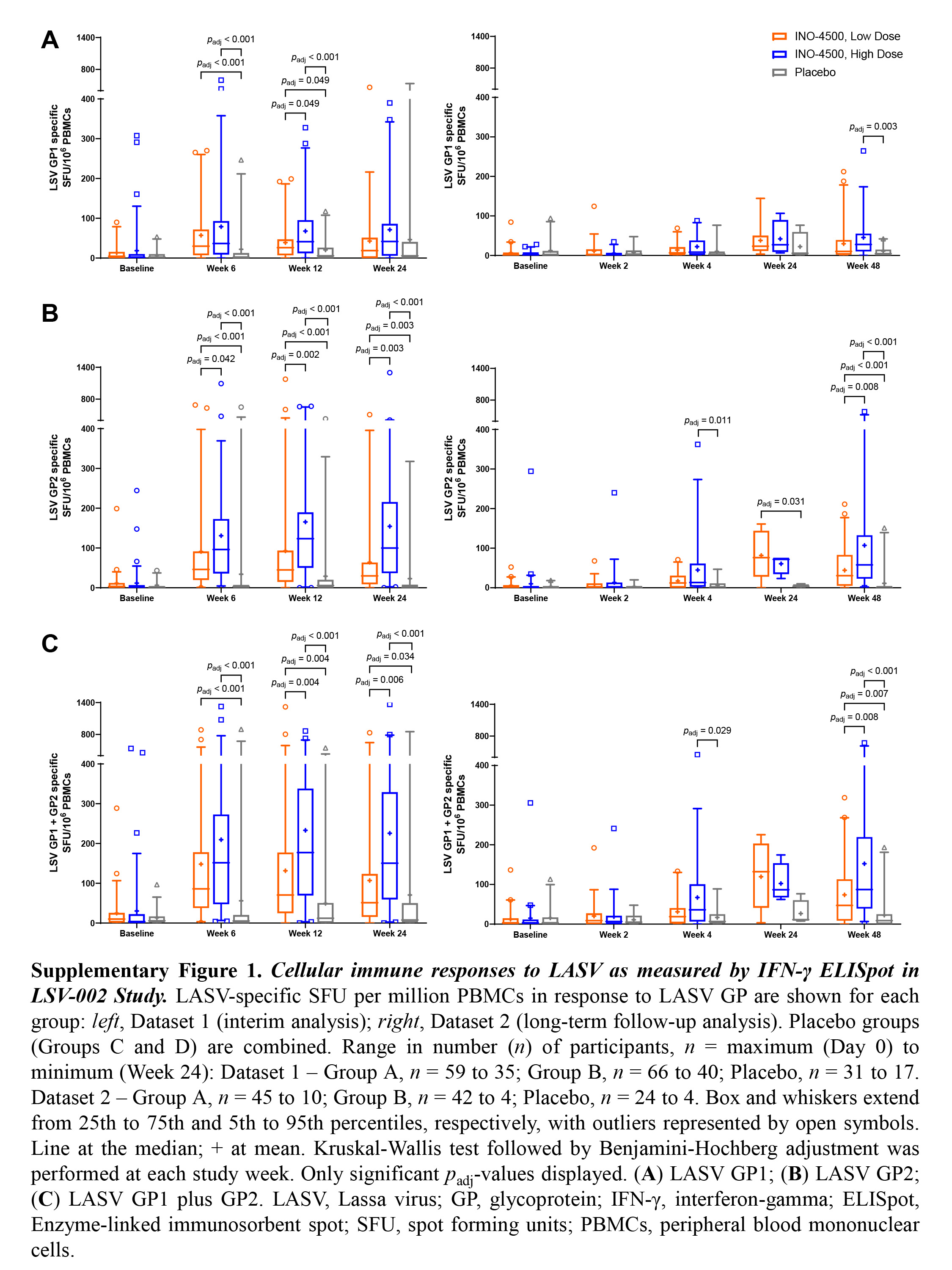

Supplement: Supplementary file 4 [file Image1.jpeg]

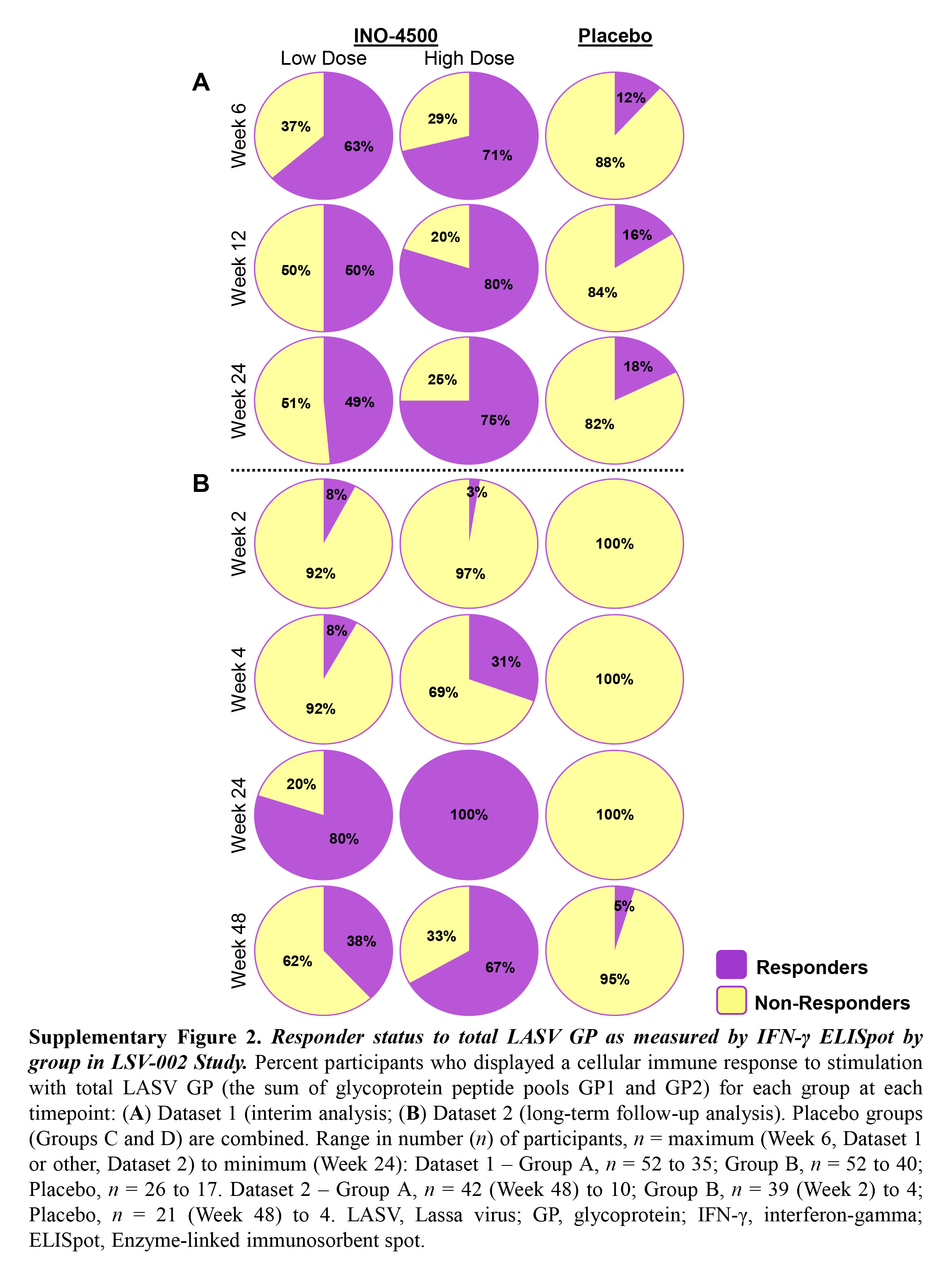

Supplement: Supplementary file 5 [file Image2.jpg]
